# A Survey of College Students on the Preference for Online Teaching Videos of Variable Durations in Online Flipped Classroom

**DOI:** 10.3389/fpubh.2022.838106

**Published:** 2022-03-09

**Authors:** Yangting Xu, Chen Chen, Dandan Feng, Ziqiang Luo

**Affiliations:** ^1^Five-Year Program of Anesthesiology, Xiangya School of Medicine, Central South University, Changsha, China; ^2^School of Biomedical Science, University of Queensland, St. Lucia, Brisbane, QLD, Australia; ^3^Department of Physiology, Xiangya School of Medicine, Central South University, Changsha, China

**Keywords:** online flipped classroom, medical education, physiology, medical student, learning preference

## Abstract

In the spring semester of 2020, online flipped classroom was used to replace offline face-to-face teaching of the physiology course at Xiangya School of Medicine. In order to analyze the preferences and utilization of different teaching resources by students, registered questionnaire was applied to investigate the preference divergence of the students on the duration of different teaching videos used in the online flipped classroom model. One hundred forty-seven students of clinical medicine in grade 2018 of Xiangya School of Medicine were selected as the research objects. Three formal surveys were conducted in total. The results showed that there were significant divergences in preference of students for different durations in the first two surveys. 56.43 and 50.00% of the students preferred 15 min mini-video, whereas 43.57 and 50.00% preferred 45 min complete video. Meanwhile, students showed a significant preference for mini-video in active learning before class, with 65.00 and 59.29% watched only mini-video, 17.14 and 25.71% watched only complete videos, and 17.86 and 15.00% watched both mini and complete videos. Although most students preferred to watch mini-video in active learning before class, there was a significant proportion of students who watched complete video before class. The results suggested that the individualization of student in the online flipped classroom is prominent. Multiple logistic regression analysis showed that the selection of videos with different durations at different time points (before, in and after class) was significantly associated with the characteristics of the videos themselves. Therefore, the construction of online teaching resources and the application of teaching methods should consider the requirements of different student groups and provide a variety of online curriculum resources.

## Introduction

In recent years, with the progressive development of educational information technology, online classes become an essential way for students to acquire knowledge with better repeatability (1) and more personalized learning methods (2). With the superiority of distance learning, online course is a new paradigm in higher education (3–5). In particular, flipped classroom teaching based on online videos becomes an important form of active learning and is rapidly spreading around the world (6). The flipped classroom reconstructs the learning process of students, in which students complete the “information transfer” by watching videos and other independent learning according to the predefined teaching objectives before the class. By understanding the learning difficulties in advance, teaching staff provide effective tutoring in the class and promote the mastering of knowledge by students through teacher-student and student-student communications (7, 8). In China, flipped classroom is widely used in medical education. Flipped classroom is very popular among students and demonstrated to improve academic performance due to its flexible learning time and increased student autonomy (9, 10).

The COVID-19 pandemic has a huge socio-economic impact globally, severely affected traditional classroom teaching structure. UNESCO recommended the distance learning and training programs and the open access platforms to reduce learning process disruption (11). As a result, online education achieved a rapid growth during the COVID-19 pandemic. For example, in China, Online education users reached 381 million in 2020 (12). Online learning is defined as “a form of distance education where technology mediates the learning process, teaching is delivered completely using the Internet, and students and instructors are not required to be available at the same time and place” (13). Available evidence suggests that online learning is at least as effective as traditional learning (14). Online Flipped Classroom (OFC) is a teaching method that combines asynchronous and synchronous online learnings (15) and is a new model for online learning in universities worldwide. It is one of the most popular educational methods during the COVID-19 epidemic and a promising alternative to teaching theoretical courses (16). Inspired by the traditional flipped classroom approach (17, 18), students were encouraged to watch video lectures (often augmented with quizzes) at home as a preparation before scientific meeting. However, unlike the traditional flipped classroom model, in OFC, students and teachers did not meet in a real classroom, but online. The time spent together was dedicated to active and collaborative learning (e.g., discussion, rather than lecturing) (19). As a widely recognized, effective, innovative, and improved strategy in the higher education of many countries, the OFC model has been recognized as an active educational approach in various fields, and more and more researchers and teachers are showing high interests in this strategy (20–22). Several studies evaluated the learning experience and learning outcomes of students in OFC, confirming that this self-directed learning model allowed students to interact with their peers or teachers, effectively stimulating their interest in learning and improving their learning efficiency (15, 19). However, we have not retrieved reports on students' preferences and needs of different instructional resources in OFC. Teaching resources directly influence the choice of teaching methods and learning experience of students, which further affects the learning efficiency of students, and ultimately manifests the learning outcomes of students (e.g., grades) (23). Therefore, investigating the teaching resources with different preference and need of students is highly beneficial to the design and construction of the teaching resources. Such investigation is an indispensable step to improve the current OFC implementation process.

Previous studies on traditional flipped classroom showed that most students prefer videos of short duration (24, 25). Students who watched short duration videos had higher participation rates and lower discrepancy compared with those watched long duration videos (26). Students showed highest attention in study when watching 10-min online instructional videos (27, 28). However, in OFC, since students and instructors did not meet physically and all teaching activities were performed online, whether students still preferred shorter duration videos is unknown.

The Department of Physiology, Xiangya School of Medicine, Central South University, recorded 72 complete videos (45–50 min/each) of traditional classroom lectures and 118 mini-videos (10–15 min/each) required for building MOOC in 2014 and 2017, both of which coverred the entire contents of physiology teaching. Both video types were taught by the same faculty teaching team. The mini-videos had the advantage of clear key points, but the disadvantage of fragmented contents. The complete videos provided a detailed and comprehensive description of the course contents, but the contents were not sufficiently highlighted and concised. The total length of mini-videos was about one-third of the total length of complete videos. Therefore, contents of one 45-min video were not equal to the contents of three 15-min videos, and they represented two completely different types of teaching resources (mini-videos were mainly on course highlights, while complete videos were more on comprehensive understanding of knowledge). Since these two different types of videos were offered in the OFC of physiology at Xiangya School of Medicine, we followed the course schedule and conducted three surveys to analyze the preferences and needs of students on the two types of instructional videos with different durations, in order to provide references for the provision and design of online instructional resources in the future.

## Materials and Methods

### Research Objects

We chose 147 students majored in 5-year clinical medicine of Grade 2018, Xiangya School of Medicine, who studied physiology by the online flipped classroom during March to April 2020. We detailed the purpose of the survey and emphasized the confidentiality principle, which was known and agreed by all participants.

### Questionnaire Design

Three formal surveys were conducted totally. The first two surveys mainly studied preference and usage of students on the teaching videos with different durations. The third survey was used to further discuss the underlying logic to the choice of students. The questionnaire was designed by the professors of department of physiology and statistician. In order to ensure the reliability of the questionnaire, 30 students were selected for the Pre-survey before the first formal survey, whose results showed that the Kronbach coefficient of the questionnaire was 0.839. The first two surveys included basic information and learning resource preferences of students. The first survey investigated the network conditions of students' participation in online learning additionally. After coding and analyzing the data, we found that the network status of students was good and the difference was not statistically significant to the content of this study. Therefore, we deleted this question and added a question about adaption of students instead. The total number of questions in first two surveys' questionnaires was 20.

### Research Methods

We used wjx (an online website for questionnaire survey) to investigate the preference of the students for different durations of teaching videos twice on 1st week and 5th week of the course. The third survey was designed based on the former results and completed on 6th week to supplement information on several notable points. The final analysis and conclusion were established through consideration of all three questionnaire surveys.

### Models of Instruction

The department of physiology of Xiangya School of Medicine conducted this online physiology teaching based on the National Quality Resource of Sharing Courses and National Quality Resource of Open Courses on the i-Course platform.

National Quality Resource of Sharing Courses http://www.icourses.cn/sCourse/course_6701.html.

Seventy two complete videos. The video content is a recording of the instructor's lecture in the classroom. The edited video lectures are from 36 to 56 min in length, averaging 46.28 min, and contain all the teaching contents of physiology. These videos are similar to the traditional face-to-face classroom, with a moderate pace of lecture, better coherence of knowledge points, teacher-student interaction, expansion and extension of relevant knowledge points, and a good learning atmosphere, which is more conducive to concentration.

National Quality Resource of Open Courses http://www.icourse163.org/course/CSU-1001930016:

One hundred eight mini-videos. The lecture content is short “micro-lectures” given on specific topics by the instructor. These deliver as voice-over Powerpoint based videos, captured and edited by a specialized online video production company in the studio. The edited video lectures are from 10 to 15 min in length, averaging 11.03 min, and contain all the teaching contents of physiology. The videos were recorded and edited to move at a fast pace. Such videos obviously do not contain time for settling in, transitions, student questions, and other activities that are part of the normal lecture. However, these videos have the advantage of prominently given to the clear key points, saving students' learning time, and having better production quality.

Specific implementation processes in OFC are as following: ① Active learn before class: students watched the videos according to the learning objectives and learning requirements released by the teacher in advance. In this process, the teacher collects learning questions from students and selects those that are commonly asked and suitable for extensive discussion and makes a list available to the QQ group before class for students to think about and prepare. ② Discuss in class: the teacher asks guided questions in the QQ group, answers queries by students, leads teacher-student interaction and student-student interaction, and helps students to master the course knowledge. ③ Review after class: students use videos and other teaching resources for independent review.

### Statistical Analysis

The data were coded, entered, and analyzed using the SPSS statistical package, version 25.0 (SPSS Inc., Chicago, IL). Descriptive statistics were performed using frequencies and percentages. The paired chi-square (χ2) test was used to compare the differences between the first and second surveys. Multiple logistic regression analysis was applied to assess the strength of association between the characteristics of two kinds of teaching videos and students' preference of watching time. A *P*-value of <0.05 was considered statistically significant.

## Results

Questionnaire surveys were carried out online and registered. The number of questionnaires collected in the first two formal surveys was 142 and 143, showing a coverage rate of 96.60 and 97.28%. Among them, 140 students filled in both first and second questionnaires were chosen to answer the third survey questionnaire; 119 students finished the questionnaire, and the coverage rate was 85.00%.

In the first survey about the quality of network for participating in online flipped classroom, students used wireless network [73.24% (104/142)], wired network [7.04% (10/142)] and mobile phone traffic [19.72% (28/142)] to watch the videos. 88.03% (125/142) students considered that network conditions were smooth enough for online flipped classroom. These results demonstrated that only 17 students thought their internet connection was not enough smooth occasionally, so the internet connection problem was excluded to influence the results.

**Figure 1:**
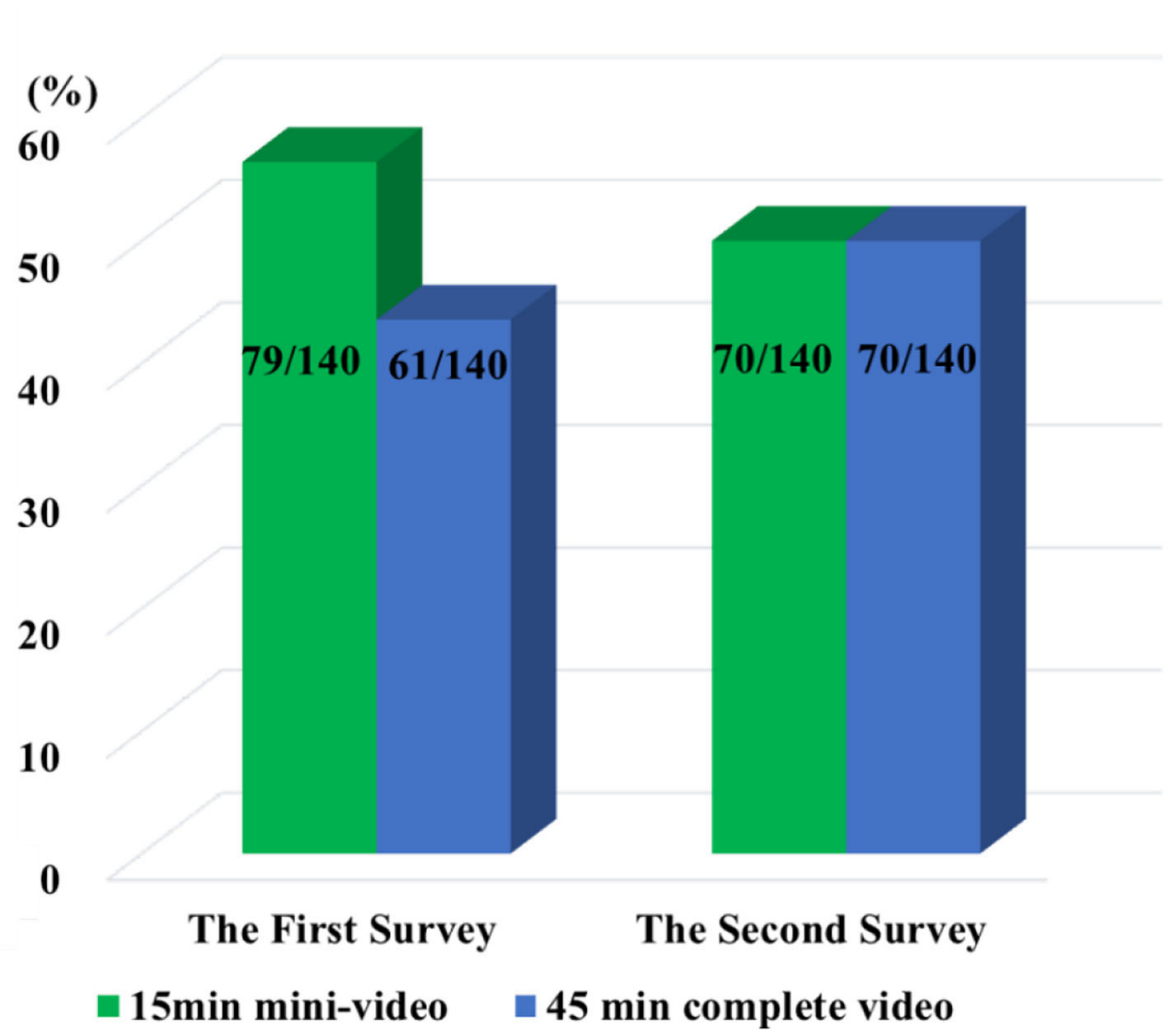
Students' preference for the teaching videos with different durations in the two surveys (*n* = 140).

In terms of preference and utilization, the percentage of students preferring 15 min mini-video in the first two surveys were 56.43% (79/140) and 50.00% (70/140), while 43.57% (61/140) and 50.00% (70/140) selected 45 min complete video (Figure 1). The paired chi-square test showed no statistical difference between the same student's preference of the teaching videos with different durations (χ^2=^ *1.641, P* = *0.200*).

Because of the huge divergence among the preference of students on the teaching videos with different durations, the third survey was devised for the logic reasons. The reasons for selecting 15 min mini-video were prominently given to the clear key points, learning time saving, more focused in watching and better video quality. Correspondingly, the reasons for selecting 45 min complete video were higher coherence of the knowledge, better learning atmosphere, expansion to connected knowledge and more focused in watching (Table 1).

**Table 1:** Percentage of students' reasons for choosing different videos.

**Reasons for students chose different video**	***n*** **(%)**
**15 min mini-video (*n* = 69)**	
Prominently given to the clear key points	64 (92.75%)
Learning time saving	42 (60.87%)
More focused in watching	33 (47.83%)
Better video quality	22 (31.88%)
**45 min complete video (*n* = 47)**	
Higher coherence of the knowledge	46 (97.87%)
Better learning atmosphere	35 (74.47%)
Expansion to connected knowledge	32 (68.09%)
More focused in watching	21 (44.68%)

**Figure 2:**
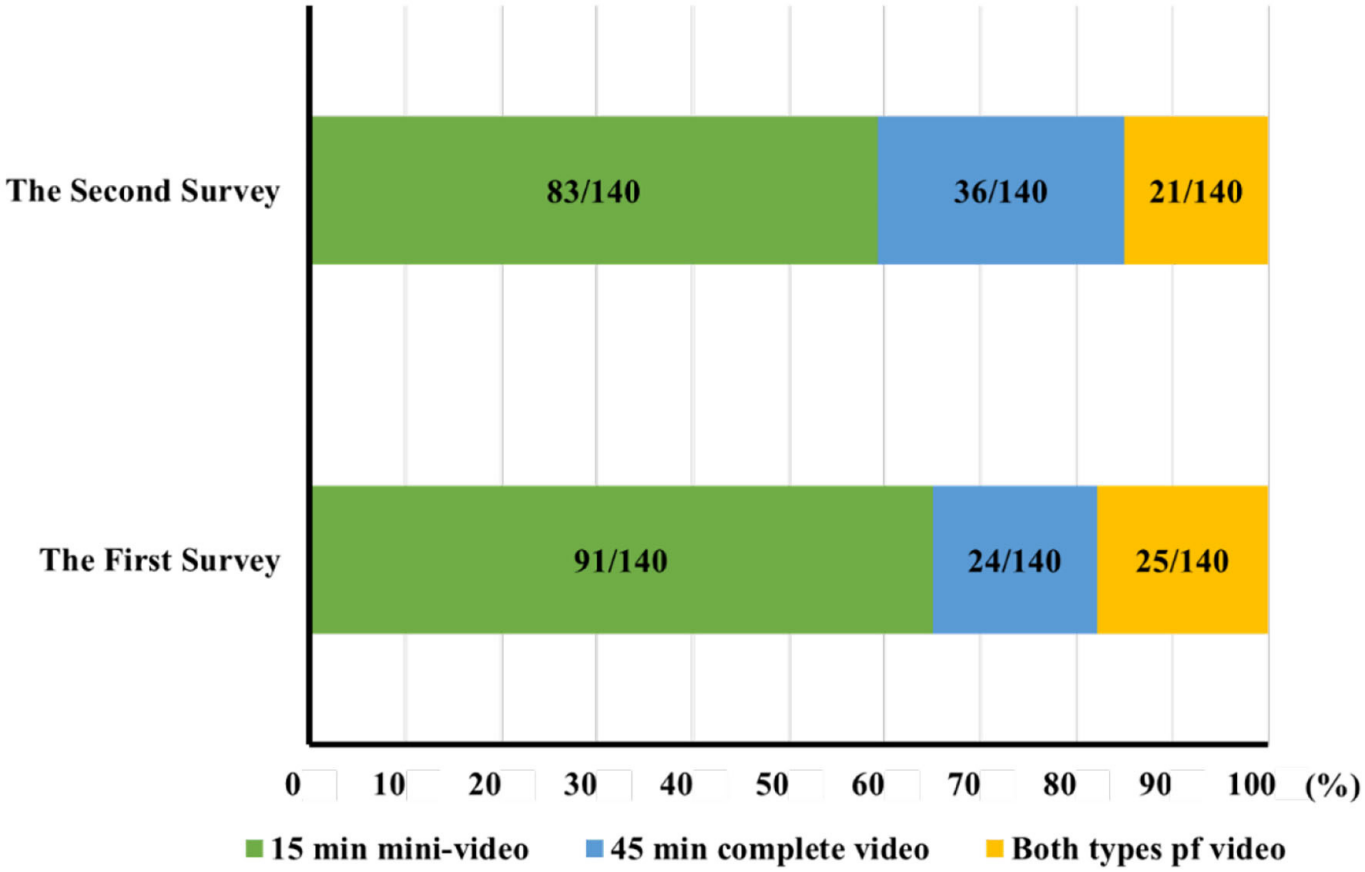
Students' preference for video types in active learning before class (*n* = 140).

To study the preference of students on teaching video in active learning before class, the answers to relative questions in the first two questionnaires were analyzed. There were 17.14% (24/140) and 25.71% (36/140) of students watched 45 min complete video and 65.00% (91/140) and 59.29% (83/140) of students watched 15 min mini-video for active learning before class; while 17.86% (25/140) and 15.00% (21/140) watched both complete and mini-video in the first two surveys (Figure 2). So 82.9% (116/140) and 74.3% (104/140) students watched 15 min mini-video before class, while 35.0% (49/140) and 40.7% (57/140) students watched 45 min complete video. Although most students preferred to watch 15 min mini-video, quite a few students preferred to watch 45 min complete video. It is obvious that different students request different types of video for active learning before class.

To further investigate the factors influencing the time (before class, in class and after class) of students watching different duration videos, we used Multiple logistic regression analysis to analyze the association between video features of two different duration videos and the watched time point (Table 2). The features including more focused in watching (χ^2^ = 1.657, *P* = 0.437), better video quality (χ^2^ = 2.197, *P* = 0.333) and time saving (χ^2 =^0.502, *P* = 0.778) had no impact on the choices of students on selecting course stage to watch 15 min mini-video. Interestingly, the feature of the prominence given key points was the factor significantly affecting the choices of students on selecting course stage to watch 15 min mini-video (χ^2^ = 8.861, *P* = 0.012). As for students that didn't endorse 15 min mini-video have the prominence given to the key points, the possibility of them to watch for active learning before class were decreased by 97% [OR = 0.03, CI = 0.01–0.41, *P* = 0.009] compare to watch for review. As for students that didn't endorse watch 45 min complete video is more focus in, the possibility of them to watch for active learning before class were decreased by 85% [OR = 0.15, CI = 0.03–0.88, *P* = 0.036] compare to watch for review. However, the features of better learning atmosphere (χ^2^ = 5.691, *P* = 0.058), expansion about other connected knowledge (χ^2^ = 1.917, *P* = 0.384), high coherence of the knowledge (χ^2^ = 4.367, *P* = 0.113) had no statistical significance in attracting students when to watch 45 min complete video.

**Table 2:** Factors influencing the time of students watching different duration videos.

**Features**	**Watch Time**	**Attitudes about Video Features**		***χ^2^***	***P***	**^†^OR (95% CI)**	***P***
		**Yes**	**No**			**No**	
		***n*** **(%)**	***n*** **(%)**				
**15 min mini-video**							
More focused in watching	Before class	25 (46.30%)	29 (53.70%)	1.657	0.437	2.50 (0.40–15.62)	0.327
	In class	4 (57.14%)	3 (42.86%)			1.10 (0.11–10.93)	0.936
	After class	4 (50.00%)	4 (50.00%)			Reference category	
The prominence given to the key points	Before class	53 (98.15%)	1 (1.85%)	8.861	0.012^*^	0.03 (0.01–0.41)	0.009^**^
	In class	6 (85.71%)	1 (14.29%)			0.26 (0.02–4.35)	0.35
	After class	5 (62.50%)	3 (37.5%)			Reference category	
Better video quality	Before class	20 (37.04%)	34 (62.96%)	2.197	0.333	0.35 (0.04–3.49)	0.373
	In class	1 (14.29%)	6 (85.71%)			1.25 (0.57–27.29)	0.888
	After class	1 (12.50%)	7 (87.50%)			Reference category	
Time saving	Before class	32 (59.26%)	22 (40.74%)	0.502	0.778	1.32 (0.23–7.62)	0.757
	In class	5 (71.43%)	2 (28.57%)			0.72 (0.08–6.84)	0.780
	After class	5 (62.50%)	3 (37.50%)			Reference category	
**45 min complete video**							
More focused in watching	Before class	15 (48.39%)	16 (51.62%)	12.348	0.002^*^	0.15 (0.03–0.88)	0.036^*^
	In class	3 (100%)	0			1.86 × 10^−9^ (0.00-∞)	0.984
	After class	3 (23.08%)	10 (76.92%)			Reference category	
Better learning atmosphere	Before class	22 (70.97%)	9 (29.03%)	5.691	0.058	7.32 (0.852–62.89)	0.070
	In class	2 (66.67%)	1 (33.33%)			1.13 × 10^−7^ (0.00-∞)	0.987
	After class	11 (84.62%)	2 (15.38%)			Reference category	
Expansion to connected knowledge	Before class	22 (70.97%)	9 (29.03%)	1.917	0.384	0.34 (0.56–2.09)	0.373
	In class	2 (66.67%)	1 (33.33%)			1.45 × 10^−6^ (0.00-∞)	0.989
	After class	8 (61.54%)	5 (38.46%)			Reference category	
Higher coherence of the knowledge	Before class	31 (100%)	0	4.367	0.113	5.53 × 10^−9^ (0.00-∞)	0.997
	In class	3 (100%)	0			3.359 × 10^−9^ (3.359 × 10^−9^-3.359 × 10^−9^)	<0.001^**^
	After class	12 (92.31%)	1 (7.69%)			Reference category	

* *P < 0.05*.

** *P < 0.01*.

† *OR (odds ratio)*.

## Discussion

This study demonstrated that the preferences of students on instructional videos with different durations in OFC were largely affected by the individual variation. In both surveys, the number of students who preferred short 15 min mini-video and those who preferred 45 min complete video were about half each. In fact, 15 min mini-video and 45 min complete video were produced by the same teaching team and were of the same quality and highly comparable. Therefore, it may be argued that the above differences in preferences do not come from the quality of video production, but rather the different students in the OFC. This result differs from the results of previous studies on traditional flipped classrooms, in which most students preferred short videos (25–28).

This study further investigated the reasons why students preferred videos with different duration. The results clearly demonstrated that the top three reasons for preferring mini-video were the clear key points, short learning time, and easy focused during study. The top three reasons for choosing complete video were higher coherence of the knowledge, better learning atmosphere, and expansion to connected knowledges. In the Pre-course active learning phase, most students tended to use mini-video, which is consistent with the findings of previous studies (25, 26). However, about one-third of the students also used complete video during Pre-course active learning. This phenomenon is strongly related to the characteristics of both types of videos. 15 min mini-video shows a clear knowledge focus. The 45 min complete video, on the other hand, may better illustrate the connection between different knowledges, and avoid the shortcoming of knowledge fragmentation in mini-video; thus may help students to understand the teaching content with depth and detail. Due to the obvious differences in preference of students on teaching videos, it is suggested that in the construction of teaching videos, it is better to provide both mini-video and complete video to students. Furthermore, the teachers should dynamically observe preference of student groups on video types during the teaching process to implement specifically targeted teaching program.

According to the Self-Determination Theory (SDT), learners can be divided into two categories: academic learners are extrinsic motivated learners in school and social learners are intrinsic motivated learners (29). The above research shows the preference of academic learners. In order to understand the preference of video duration by social learners, we uploaded these two durations of videos for the society on Bilibili (a Chinese website for watching online videos) (mini-video website https://www.bilibili.com/video/BV1xW411q7jm?from=search, complete video website https://www.bilibili.com/video/BV1Et411D7qf?from=search). The users can play these videos freely, so the view count number of the video has the capacity to reveal the social preference for each type of videos. The average view counts of each video per month were collected for statistical analysis. Up to July 20th, 2020, when the spring semester came to an end, the count of 15 min mini-video was 73.89 and the count of 45 min complete video was 245.30, showing obvious difference. It is therefore suggested that the social learners prefer to watch 45 min complete video to satisfy their learning requirement. Since academic learners and social learners have different learning motivations, the huge differences in the preference of video duration between the two types of learners may be related to the difference in motivation. It is suggested that the learning motivation may play a critical role in the preference for the duration of instructional video. The above results suggest that student groups with different learning motivations may have completely different preferences for instructional resources. Teaching staff should consider different motivation groups when building a library of teaching resources. It is suggested that the teaching resources should be enriched, and both short-term intensive lecture resources and complete teaching videos which was similar to traditional classrooms contents should be provided to meet the needs of people with different learning motivations.

## Conclusions

This report demonstrates the differences in preferences of students on different lengths of learning videos in the online flipped classroom. Through analysis, the different features of the 15 min mini-video and the 45 min complete video may be a determining factor leading to the significant difference in preferences of students. Based on the significant difference presented by the student groups in this study, it is suggested that only providing 15 min mini-video or 45 min complete videos will not sufficiently meet the best educational demand. At the same time, the huge difference in the video preferences by different learning motivation groups suggests that the learning motivation may become an important factor determining preference of students on instructional videos.

This report reminds that the provision of online teaching resources and the design of teaching methods should base on different education stages and purposes. The needs of different student groups should be considered. In order to improve the quality of online teaching, a rich library of teaching video online resources of different durations and contents should be constructed.

## Data Availability Statement

The original contributions presented in the study are included in the article/supplementary material, further inquiries can be directed to the corresponding authors.

## Ethics Statement

The studies involving human participants were reviewed and approved by Ethics Committee of Central South University. The patients/participants provided their written informed consent to participate in this study.

## Author Contributions

YX: questionnaire survey, data sorting, statistical analysis, and thesis writing. DF: questionnaire design and thesis writing. CC and ZL: research guidance, paper conception, and revision. All authors contributed to the article and approved the submitted version.

## Funding

This work was supported by Hunan Province colleges ideological and political courses construction project (HNKCSZ-2020-0061), Hunan Province academic degree and graduate education reform project (2020JGYB028), Research on deepening innovation and entrepreneurship education reform of Central South University (2020jy139), Central South University Ideological and Political Courses construction project (2019096).

## Conflict of Interest

The authors declare that the research was conducted in the absence of any commercial or financial relationships that could be construed as a potential conflict of interest.

## Publisher's Note

All claims expressed in this article are solely those of the authors and do not necessarily represent those of their affiliated organizations, or those of the publisher, the editors and the reviewers. Any product that may be evaluated in this article, or claim that may be made by its manufacturer, is not guaranteed or endorsed by the publisher.
